# Colonization of *Lutzomyia verrucarum* and *Lutzomyia longipalpis* Sand Flies (Diptera: Psychodidae) by *Bartonella bacilliformis*, the Etiologic Agent of Carrión’s Disease

**DOI:** 10.1371/journal.pntd.0004128

**Published:** 2015-10-05

**Authors:** James M. Battisti, Phillip G. Lawyer, Michael F. Minnick

**Affiliations:** 1 Division of Biological Sciences, University of Montana, Missoula, Montana, United States of America; 2 Laboratory of Parasitic Diseases, National Institutes of Allergy and Infectious Diseases, National Institutes of Health, Bethesda, Maryland, United States of America; Lancaster University, UNITED KINGDOM

## Abstract

*Bartonella bacilliformis* is a pathogenic bacterium transmitted to humans presumably by bites of phlebotomine sand flies, infection with which results in a bi-phasic syndrome termed Carrión’s disease. After constructing a low-passage GFP-labeled strain of *B*. *bacilliformis*, we artificially infected *Lutzomyia verrucarum* and *L*. *longipalpis* populations, and subsequently monitored colonization of sand flies by fluorescence microscopy. Initially, colonization of the two fly species was indistinguishable, with bacteria exhibiting a high degree of motility, yet still confined to the abdominal midgut. After 48h, *B*. *bacilliformis* transitioned from bacillus-shape to a non-motile, small coccoid form and appeared to be digested along with the blood meal in both fly species. Differences in colonization patterns became evident at 72h when *B*. *bacilliformis* was observed at relatively high density outside the peritrophic membrane in the lumen of the midgut in *L*. *verrucarum*, but colonization of *L*. *longipalpis* was limited to the blood meal within the intra-peritrophic space of the abdominal midgut, and the majority of bacteria were digested along with the blood meal by day 7. The viability of *B*. *bacilliformis* in *L*. *longipalpis* was assessed by artificially infecting, homogenizing, and plating for determination of colony-forming units in individual flies over a 13-d time course. Bacteria remained viable at relatively high density for approximately seven days, suggesting that *L*. *longipalpis* could potentially serve as a vector. The capacity of *L*. *longipalpis* to transmit viable *B*. *bacilliformis* from infected to uninfected meals was analyzed via interrupted feeds. No viable bacteria were retrieved from uninfected blood meals in these experiments. This study provides significant information toward understanding colonization of sand flies by *B*. *bacilliformis* and also demonstrates the utility of *L*. *longipalpis* as a user-friendly, live-vector model system for studying this severely neglected tropical disease.

## Author Summary

In general, *Bartonella* infections are zoonooses and are transmitted to humans by hematophagous (blood eating) arthropods such as fleas, lice, ticks, mites and sand flies. *Bartonella bacilliformis* is found only in Andean regions of South America and is transmitted to humans by a few select species of sand flies, resulting in Carrión’s disease. Our current understanding is that Carrión’s disease is not a zoonosis, as an animal reservoir has not been identified. In this study, we analyzed *B*. *bacilliformis* colonization in two species of sand flies in an attempt to understand why *Lutzomyia verrucarum* is a competent vector and *Lutzomyia longipalpis* is incapable of transmitting this pathogen between humans. We found that *B*. *bacilliformis* remains in the abdominal midgut of the non-competent vector and is progressively digested until no viable bacteria remain (7d). Furthermore, large groups of *L*. *longipalpis* were used to demonstrate that this non-competent species is incapable of mechanically transmitting viable bacteria between artificial blood meals simply by contaminated mouthparts. In *L*. *verrucarum*, *B*. *bacilliformis* colonizes the lumen of the digestive tract beyond the intra-peritrophic space in large numbers and persists (>14d).

## Introduction

*Bartonella bacilliformis*, the focus of this study, is the bacterial agent of a potentially life-threatening bi-phasic disease referred to as Carrión’s disease. Known since pre-Incan time, estimates of human fatalities caused by *B*. *bacilliformis* are >100,000 [1]. Since the first known epidemics in 1871, this neglected tropical disease continues to impose significant morbidity and mortality on South Americans; approximately 1.7 million of which are currently estimated to be at risk [2]. The *B*. *bacilliformis* ecotype [3,4] is limited to South American humans and select species of sand flies living in the Andes Mountains of Peru, Ecuador and Colombia.

Because a nonhuman reservoir has not been identified, our current understanding is that *B*. *bacilliformis* is transmitted among humans by phlebotomine sand flies. *Bartonella bacilliformis* proliferates in the human bloodstream by invading erythrocytes, and 2–8 weeks following the bite of an infected sand fly, resulting in an illness characterized by hemolytic anemia and concurrent Oroya Fever with fatality rates as high as 90% [1]. If an individual survives this primary anemic phase, verruga peruana (VP) or Peruvian warts may develop. These verrucous skin eruptions result from *B*. *bacilliformis’* capacity to invade vascular endothelial cells and stimulate angiogenesis [5,6] [7]. VP are hemangioma-like cutaneous microcolonies, histologically indistinguishable from bacillary angiomatosis (BA) lesions of individuals infected with *Bartonella henselae* or *Bartonella quintana* [8].

Phlebotomine sand flies are responsible for transmitting a number of pathogens causing diseases in humans, including leishmaniasis, viral encephalitis and Carrión’s disease. “Sand fly” is a term derived from the tan or sandy color of the wings of many of the >800 species described to date [9]. In general, the sand fly life cycle is rather long, requiring 45–80 d to complete all developmental stages, including egg, four larval instars, pupa and adult. Plant nectar likely provides a significant source of nutrients and water for both male and female flies, but only females are hematophagous [10].

It is well established that *L*. *longipalpis* transmits *Leishmania* to humans, yet for unknown reasons this species does not seem capable of serving as a vector of *B*. *bacilliformis*. This hypothesis is based largely on the apparent lack of *B*. *bacilliformis* in naturally-occurring populations of *L*. *longipalpis* or any other arthropod sampled in endemic areas. For example, Noguchi’s group (late 1920’s) tested a large number of arthropods from endemic ‘verruga zones’ by homogenization, injection into animals, and subsequent isolation. Largely based on these results, *L*. *verrucarum*, *Lutzomyia peruensis* and *Lutzomyia noguchi* are considered competent sand fly species associated with *B*. *bacilliformis* transmission to humans [11].

“Vector specificity” is a term used to describe the phenomenon where one species of arthropod such as *L*. *verrucarum* is capable of transmitting a particular pathogen, like *B*. *bacilliformis*, and another closely-related species inhabiting the same ecotype such as *L*. *longipalpis* is not [12]. Thus, we asked if the apparent vector specificity of *B*. *bacilliformis* exhibited between *L*. *verrucarum* (competent) and *L*. *longipalpis* (non-competent) was the result of each fly’s blood meal preference or whether a developmental relationship between pathogen and competent vector has evolved.

In this study, we analyzed several aspects of *B*. *bacilliformis’* interactions with *L*. *verrucarum* and *L*. *longipalpis* sand fly species. First, we transformed a low-passage *B*. *bacilliformis* isolate to synthesize green fluorescent protein (GFP). Second, we used this GFP^+^ strain to artificially infect *L*. *verrucarum* and *L*. *longipalpis* sand flies in order to observe bacterial colonization of both species over a 14-d time course by fluorescence microscopy. Third, we artificially infected *L*. *longipalpis* and assessed the viability of *B*. *bacilliformis* in adult flies, eggs, feces and diuretic fluid. Finally, we assessed the capacity of *L*. *longipalpis* to transmit *B*. *bacilliformis* between “infected” and “un-infected” artificial blood feeders. This is the first report analyzing *B*. *bacilliformis* colonization of *L*. *longipalpis*, subsequent viability and potential for transmission by this sand fly species.

## Materials and Methods

### Ethics Statement

All experiments involving animals were approved by the University of Montana Institutional Animal Care and Use Committee under protocol number 063-12-MMDBS-010213. The specific regulations to which this animal care and use protocol adhered was The National Resource Council’s Guide for Care and Use of Laboratory Animals (8^th^ edition). The University of Montana is also accredited by AAALAC with PHS assurance and is currently registered with the USDA.

### Bacterial Cultivation and Genetic Manipulation

*Bartonella bacilliformis* was cultivated as previously described for *B*. *quintana* except the incubation temperature was 30°C and 5% CO_2_ was omitted [13]. Briefly, heart infusion broth + sheep blood (HIB-B) plates consisted of heart infusion broth containing 4% sheep blood, 2% sheep serum and 1.5% agar. When necessary, HIB-B was supplemented with 25 μg/ml kanamycin (HIB-B+K). *Bartonella bacilliformis* was routinely cultured 5–7 d, harvested using a flat razor, washed (3 times with PBS, pH7.4) and added to freshly prepared human blood (below).

*Bartonella bacilliformis* [strain San Pedro] was transformed by electroporation with pJMB-GFP as previously described [14]. The resulting low-passage GFP^+^ strain of *B*. *bacilliformis* was used for all experiments in the study, and for simplicity will be referred to as *B*. *bacilliformis* throughout the text.

### Sand Fly Mass Rearing

All manipulations were performed in a dedicated sand fly insectary equipped with an air pressurized vestibule entry room (University of Montana). The *L*. *longipalpis* strain, which originated from Jacobina, Brazil, was obtained from an existing colony maintained at the Walter Reed Army Institute of Research (WRAIR, BEI Resources, Catalog No. NR-44001). One of the authors (PGL) initially isolated the *L*. *verrucarum* strain used in the study from case sites in the vicinity of Caraz, Ancash Department, Peru, and subsequently established a colony at WRAIR. To our knowledge, this is the only laboratory colony of *L*. *verrucarum* currently in existence. *Lutzomyia longipalpis* is much more amenable to mass rearing and manipulation in the laboratory than is *L*. *verrucarrum*, which requires a lower rearing temperature, has a longer generation time and lower fecundity and productivity. For this reason, *L*. *longipalpis* was used for the majority of this study as we did not want to risk sacrificing the rare *L*. *verrucarum* laboratory colony.

Flies were maintained according to standard mass-rearing procedures developed and implemented at the WRAIR [15] [16]. Further details regarding maintenance and mass rearing of flies can be found in supplementary information (S1 Data).

### Artificially Infected Human Blood Meals

Fresh human blood (Type O^+^) was collected from one of the authors (JMB) using anticoagulant acid-citrate-dextrose solution B (BD Vacutainer 364816; Becton Dickenson, Franklin Lakes, NJ), and immediately transferred to 40°C. Cells were separated from serum by centrifugation (1000xg, 5min, 4°C) washed three times (PBS, pH7.4, 4°C) and reconstituted with heat-inactivated serum (56°C, 1h) from a Type O^+^ blood donor. *Bartonella bacilliformis* (strain San Pedro) harboring pJMB-GFP was cultured on HIB-B+K for 5–7 d. Bacteria and erythrocytes were enumerated by light microscopy, combined with heat-inactivated serum prepared as above (at an MOI of ~10 bacteria per RBC) and co-cultured at 30°C for either 3 h or 16 h.

Five-d-old frozen chicks (Layne Laboratories, Arroyo Grande, CA) were thawed in PBS and skins carefully removed. Skins were sterilized by soaking for 1 min in 70% EtOH, rinsed three times in sterile PBS and mounted on custom-made glass membrane feeders (Lillie Glassblowers; Atlanta GA; autoclaved) with orthodontic rubber bands and dental wax. After testing the membrane for leaks using PBS, the blood meal was loaded into the glass feeder, and the feeder sealed with parafilm, warmed with a circulating water bath (39°C) and offered to flies housed in 473-ml experimental feeding cups [Neptune paper cans (WL Enterprises Inc, Fort Lee, NJ)] modified in two ways. Feeding cups were prepared as follows: First, a 2.45-cm diameter aspirator access door was covered with a double-layer of dental dam (Coltene Whaledent; Cuyahoga Falls, OH) and was taped to the cup with strapping tape. Second, cup lids were replaced with CM and held in place with strapping tape allowing flies access through the fabric to soaked cotton balls as well as artificial blood meals. Flies in experimental cups were stored in polypropylene boxes as described above for larval pots (25°C; 80–90% RH).

Starvation of flies for 24 h increased feeding activity and sucrose-soaked cotton was replaced with water-soaked cotton 16h prior to (and during) artificial blood meals. After feeding on artificial blood meals, flies were fed a 10% sucrose solution supplemented with 40 mg/ml kanamycin (by cotton) and stored as above (25°C; 80–90% RH). At 16–32h following the blood meal, flies that had obviously imbibed blood (distinguished by dark color of abdomen; typically ~33–50% of the population) were transferred to new experimental cups for further analysis. As an additional safety measure, cups containing infected flies were stored in polypropylene boxes and sealed in air-tight clear polyethylene bags (Ziploc; C. Johnson & Son, Racine, WI).

### Microscopic Examination of *B*. *bacilliformis* Colonization of *L*. *verrucarum* and *L*. *longipalpis*

Infected blood meals were prepared and individually fed to small populations (~150 females) of *L*. *verrucarum* and *L*. *longipalpis*. At 1, 2, 3, 5, 7, and 14 d (*L*. *verrucarum* only at 14d) following ingestion of infected blood meals, three individual flies of each species were dissected and analyzed for the presence of *B*. *bacilliformis* using UV-fluorescence microscopy. In addition to the digestive tract, all other tissues and organs of the flies were examined, including hemolymph, salivary glands, mouthparts and crop. Eggs, feces, and diuretic fluid generated by the infected flies were also analyzed for *B*. *bacilliformis* by fluorescence microscopy.

Blood-fed females were transferred by mouth aspirator (JW Hock; Gainesville, FL) from experimental cups to sterile, 15-ml screw-cap conical tubes containing water and one drop of liquid dish soap. Flies were gently puffed from the aspirator into the tube and were immobilized in the bubbles. Tubes containing infected flies were capped, gently inverted and poured onto a 10-cm^2^ cloth mesh (CM) suspended over a standard Petri dish. Soap was rinsed away by moving the CM to Petri plates containing water or 1X PBS. As an additional safety precaution, all manipulations of living, infected flies were performed in a custom-made glove box within the insectary.

**Fig 1:**
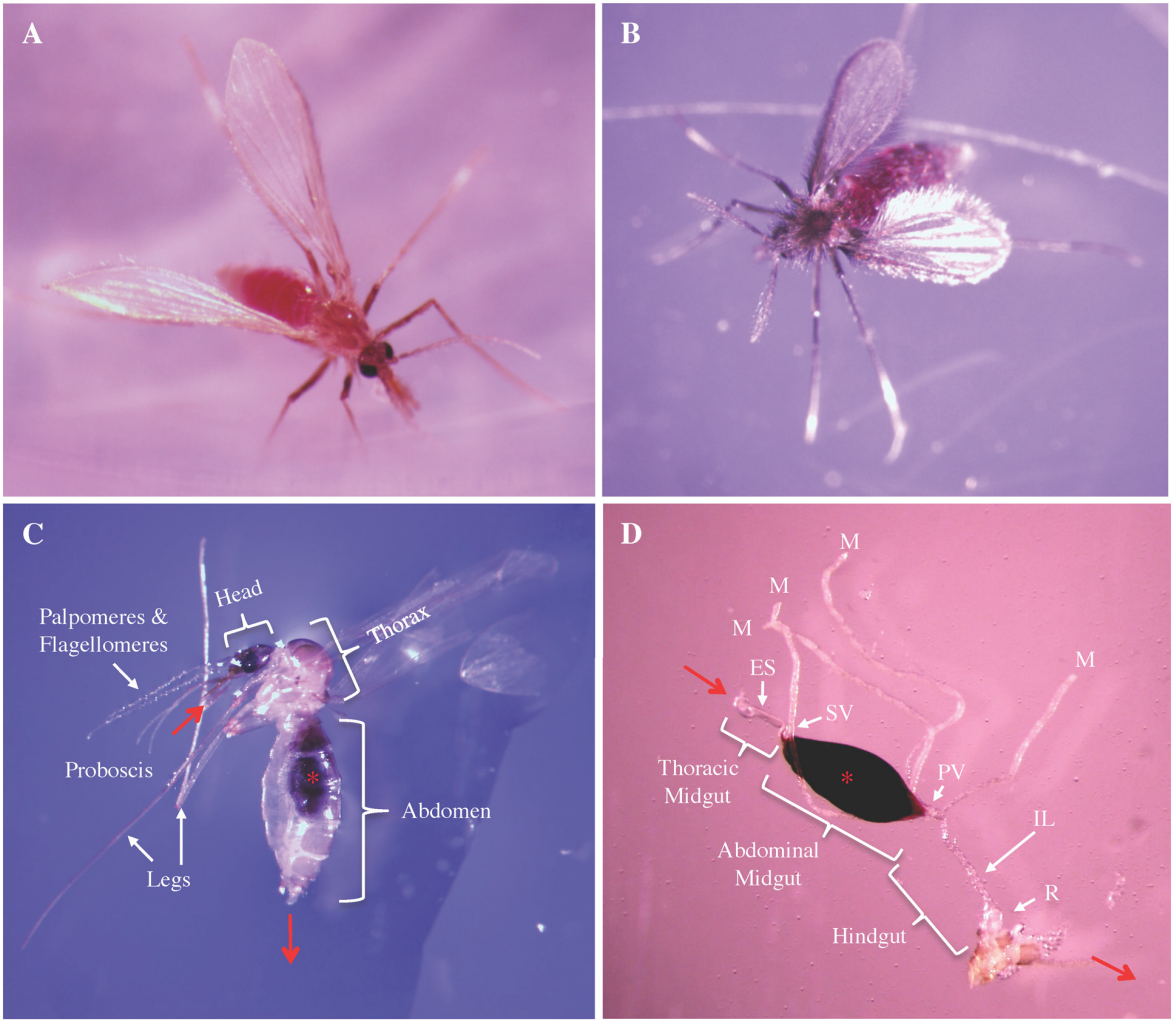
Anatomy of female *Lutzomyia* sand flies. *L*. *verrucarum* **(A)** and *L*. *longipalpis* **(B)** 16h following ingestion of a *B*. *bacilliformis-*infected human blood meal. (**C**) External anatomy of *L*. *verrucarum* with red arrows illustrating route of blood meal from probosis through head, thorax, and abdomen. (**D)** Blood-fed *L*. *verrucarum* 24h following ingestion of an artificial blood meal * which is held within the abdominal midgut (AM) by a peritrophic membrane (PM) between the stomodeal valve (SV) and pyloric valve (PV). Flies digest blood meal by releasing enzymes into the PM-encased lumen of the AM. Esophagus (ES), Ileum (IL), Rectum (R), Malpighian tubules (M).

Subsequent manipulation and dissection were accomplished using a stereoscopic microscope (StereoZoom; Bausch & Lomb) and wooden applicator sticks (15 cm long, 0.32 cm dia.) tipped with stainless steel entomology needles (Minuten Nadeln, BioQuip, Rancho Dominguez, CA). After transferring individual flies to a 50-μl drop of PBS on a microscope slide (Fig 1C), one needle was used to hold the fly (by piercing thorax), and the other was used to remove the head and pull the entire alimentary tract and attached organs away from the exoskeleton. This structure was subsequently transferred to a 20-μl drop of PBS on a separate microscope slide and then covered with a coverslip. The gut contents were examined using a microscope (BX51; Olympus, Center Valley PA) equipped with a fluorescence illuminator (X-Cite 120Q; Excelitas Technologies, Waltham MA) and a cooled digital color camera (DP72; Olympus) with accompanying acquisition software (DP2-BSW; Olympus).

### Viability *of B*. *bacilliformis* in *L*. *longipalpis* Adult Flies, Eggs, Feces and Diuretic Fluid

Four groups of 200 female flies each were transferred from adult cages to experimental cups using a custom, vacuum-assisted transfer device. Cups contained flies of known ages; two cups contained ‘young’ flies (2-10-d-old) and the other two contained ‘old’ flies (11-25-d-old). An infected blood meal was prepared as described above (i.e., bacteria and blood co-cultured for 16h at 30°C), loaded into four individual glass feeders fitted with sterilized chick-skin membranes and warmed with circulating water (15 min at 39°C). Cups containing flies were placed under each of the four identical feeders loaded with infected blood meals and flies were allowed to feed (3 h, 80–90% RH). Cups containing infected flies were then stored (32 h, 25°C; 80–90% RH) to allow for diuresis and hardening of peritrophic membranes, after which flies from each group that had imbibed blood were transferred to new experimental cups (one cup per group) and stored for further examination.

At days 3, 5, 7, 9, 11, and 13 following ingestion of an infected blood meal, individual flies from ‘old’ and ‘young’ populations were examined for viable *B*. *bacilliformis* colony forming units (CFUs). At each time point, five flies were transferred from each age group to two tubes containing PBS (with dish soap as above). Each tube of infected flies was gently inverted and contents poured into a sterile cell strainer (70 μM, #352350; BD Falcon, Bedford, MA) and placed in a sterile Petri plate.

In general, surface sterilization of the infected flies was accomplished as previously described [17]. Cell strainers containing flies were then transferred to 15-ml sterilization solution (70% ethanol, 0.05% sodium hypochlorite) swirled in a Petri dish on a level bench top (45 s), washed (5 ml PBS; 3 times for 1 min each), and plated as follows: At each time point, eight surface-sterilized flies (four from each age group) were individually transferred to 1.5-ml microcentrifuge tubes (each containing 25 μl PBS), homogenized with sterile pestles (Argos Technologies; Elgin, IL), serially diluted in heart infusion broth and plated on HIB-B+K.

Eggs, feces, and diuretic fluids generated by infected flies were also examined for *B*. *bacilliformis*. At 8-10d following infection, eggs, feces and diuretic fluids were collected from experimental cups, serially diluted and plated onto HIB-B+K. Specifically, a micropipettor and 10-μl aliquots of PBS were used to randomly collect four groups of five eggs each that were surface-sterilized, washed, homogenized, diluted and plated as above for adult females.

Diuretic fluid and feces generated by *L*. *longipalpis* following the blood meal were also sampled for viable bacteria. Four batches of five piles each of randomly collected feces and four batches of five dried diuresis droplets each were independently collected using a micropipettor and aliquots of PBS. These samples were serially diluted (HIB) and plated (HIB-B+K). Following a 30-d incubation at 30°C, *B*. *bacilliformis* CFUs were counted.

### Mechanical Transmission of *B*. *bacilliformis* by *L*. *longipalpis*.

Two groups of female flies were transferred from adult cages to experimental cups using a vacuum aspirator. Two to five-d-old flies were used in this transmission study, and cups contained different numbers, either 50 or 150 female *L*. *longipalpis*. These two groups of flies were starved overnight and then individually assessed for their capacity to transmit viable *B*. *bacilliformis* from an infected human blood meal to an uninfected meal in the membrane feeder.

Three blood meals were prepared as above except only one contained *B*. *bacilliformis* (~6x10^6^ bacteria per ml). Each group of flies was first offered the infected blood meal and then offered an uninfected blood meal specific to each group. Infected and uninfected artificial meals were offered in three successive trials of 5, 10, and 15 min each. For example, the group of 50 flies was offered an infected blood meal for 5 min and then this group of flies was moved to an uninfected meal for 5 min. This same sequential (infected then uninfected) method was repeated two additional times for durations of 10 min and then 15 min with the group of 50 flies. The group of 150 flies was also independently fed this same infected meal in an identical manner and a separate, uninfected blood meal was used for this group. Contents of both uninfected feeders were then plated and cultured as above. Following a 30-d incubation at 30°C, *B*. *bacilliformis* CFUs were enumerated.

## Results

### Microscopic Examination of *B*. *bacilliformis* Colonization of *L*. *verrucarum* and *L*. *longipalpis*

In initial pilot experiments, we observed significant differences in colonization results between flies fed meals that had been co-cultured for 3h or 16h. In both species of sand fly, colonization was significantly lower in flies fed 3-h co-cultures as compared to 16 h. Flies examined by fluorescence microscopy at 3 and 5 d post-blood meal had relatively little GFP signal when fed 3-h co-cultures, whereas flies of both species that had imbibed 16-h co-cultured blood meals had obvious colonization that was of markedly higher density. For this reason, 16-h co-cultures were used for the remainder of the study.

**Fig 2:**
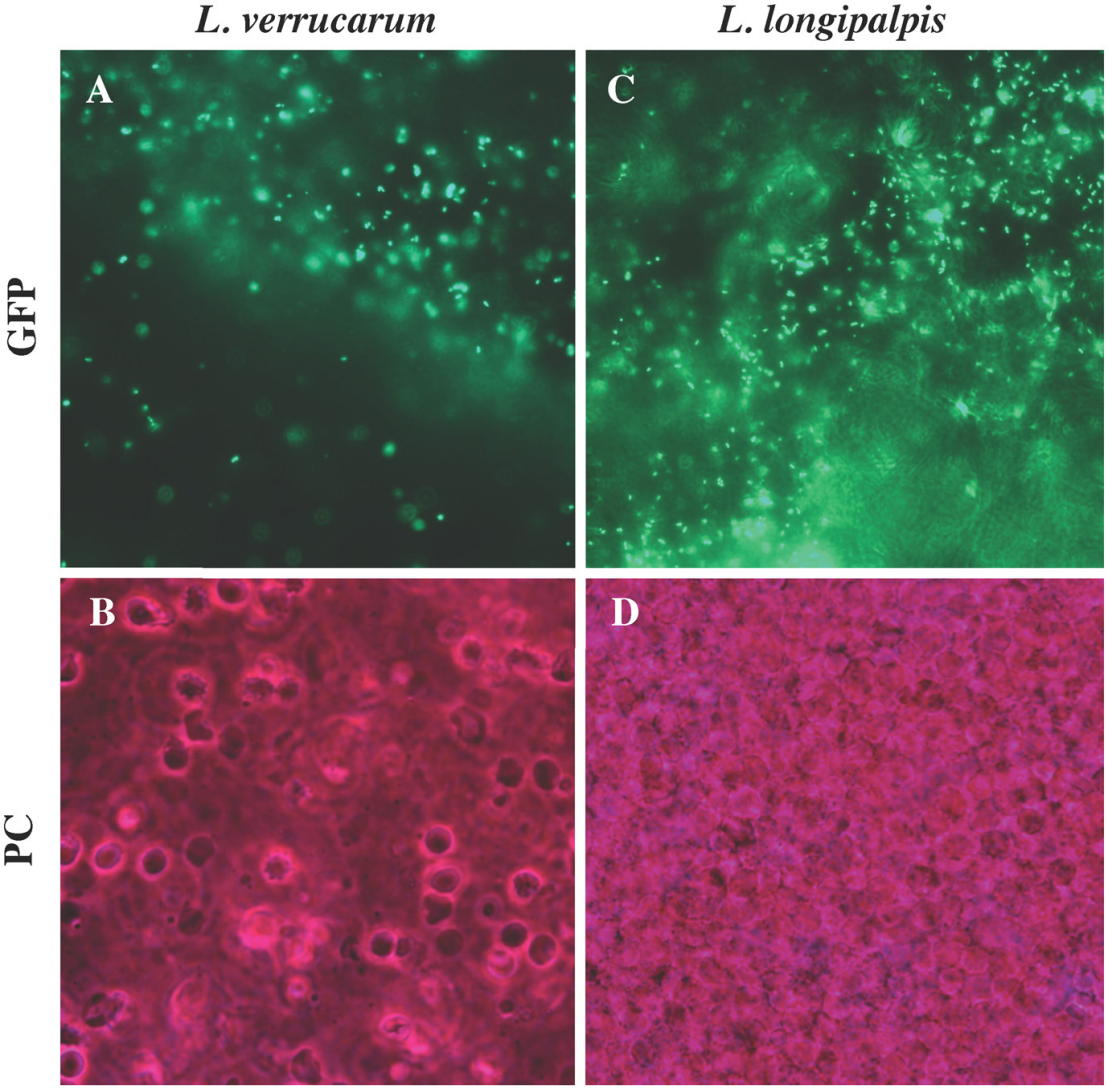
*B*. *bacilliformis* colonization of sand flies 24h following ingestion of infected blood meal. *B*. *bacilliformis* was highly concentrated in the abdominal midgut 24h following ingestion of an infected blood meal, colonization of *L*. *verrucarum* (**A-B**; magnified 1000x) and *L*. *longipalpis* (**C-D;** magnified 400x) was indistinguishable. At this time point, colonization was limited to abdominal midgut and bacteria were not observed in any other sand fly tissue. Image pairs were acquired with fluorescence (GFP) or phase contrast (PC) microscopy. *B*. *bacilliformis* exhibited a marked degree of motility in both species of sand fly (*see* S1 and S2 Videos).

Microscopic examination of sand fly tissues included salivary glands, haemocoel, mouth parts, eggs, feces, and diuretic fluids and were performed over a 14-d time course. At 24h post-blood meal, *B*. *bacilliformis* was found highly concentrated in the abdominal midgut (AM) of both *L*. *verrucarum* and *L*. *longipalpis* (Fig 2). Bacteria were confined to this region of the digestive tract by the stomodeal and pyloric valves and were enveloped by the peritrophic membrane (PM), where all bacteria were held in the lumen of the AM. Bacteria were not found in any other tissue of either fly species at this time point.

At 24h post-blood meal, bacteria were highly motile and bacillus-shaped, measuring approximately 0.5–1 um wide and 1–3 um long. Although bacteria demonstrated a marked degree of motility in the AM of *L*. *verrucarum* (S1 Video) and *L*. *longipalpis* (S2 Video), *B*. *bacilliformis* appeared to adhere to the internal surface of the PM of both sand fly species. At 24h following an infected blood meal, differences in bacterial colonization of *L*. *verrucarum* and *L*. *longipalpis* flies were indistinguishable.

**Fig 3:**
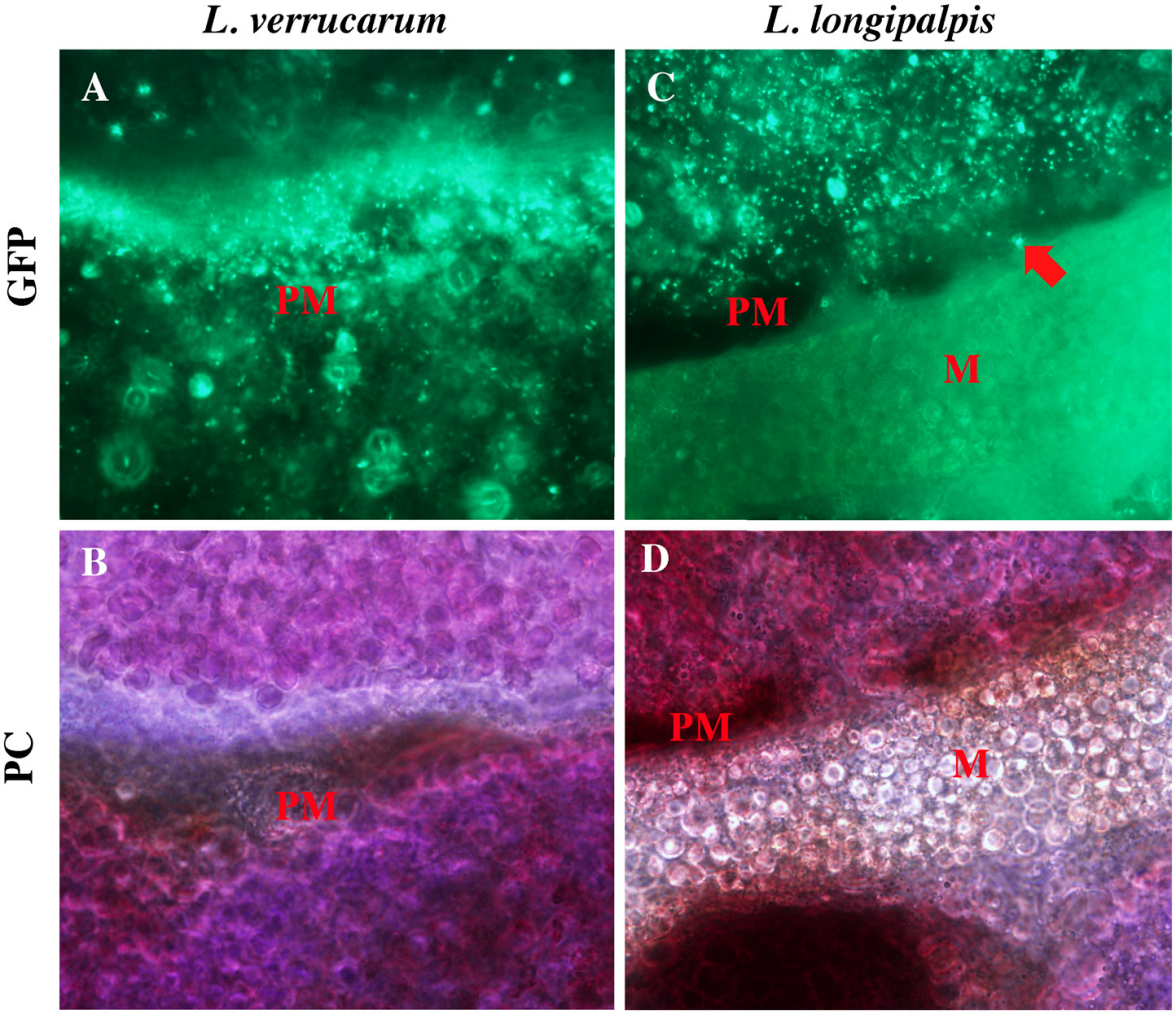
*B*. *bacilliformis* colonization of sand flies 48h post-blood meal. At this time point, colonization of *L*. *verrucarum* (**A-B**) and *L*. *longipalpis* (**C-D**) was indistinguishable. Bacteria appeared to adhere to peritrophic membrane (PM) and although colonization was limited to abdominal midgut (shown here) on rare occasion, appeared outside the PM (arrow). The majority of bacteria changed morphology into non-motile small coccoid forms (*see* S3 Video), and appeared more numerous than at 24h. Images acquired (1000x) with fluorescence (GFP) or phase contrast (PC) microscopy.

At 48h post-blood meal, bacteria appeared more numerous (as compared to the 24-h time point) and had changed their morphology to smaller coccoid forms of approximately 1 μm in diameter (Fig 3). On rare occasions, bacteria were observed on the apparent external side of the PM and one of the *L*. *verrucarum* sampled at this time point showed a small number of bacteria in the esophagus. In addition to changing shape, motility was significantly decreased and bacteria appeared adherent to the inner wall of the PM (S3 Video). At 48h, colonization of both fly species by *B*. *bacilliformis* appeared the same.

**Fig 4:**
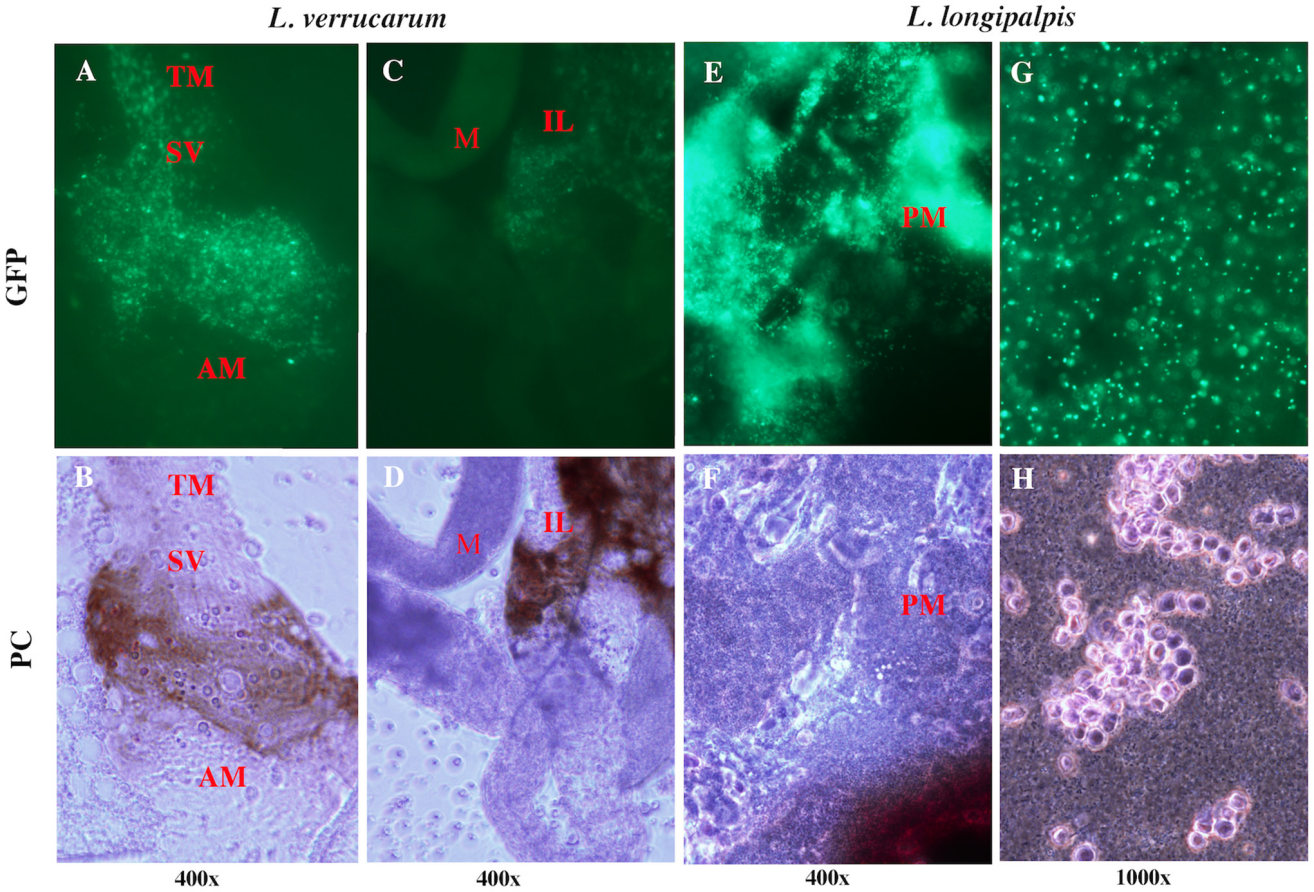
72h post-blood meal. At this time point, colonization of the fly species appeared different, where *B*. *bacilliformis* were observed at relatively high density in locations outside the abdominal midgut of *L*. *verrucarum*, but not *L*. *longipalpis*. *B*. *bacilliformis* was found in the thoracic midgut **(A-B)** as well as the ileum **(C-D)** of *L*. *verrucarum*. *B*. *bacilliformis* colonization of *L*. *longipalpis* was limited to the abdominal midgut (**E-H**) and bacteria appeared to be digested along with the blood meal. Image pairs were acquired with fluorescence (GFP) or phase contrast (PC) microscopy.

At 72h post-blood meal, digestion of erythrocytes in the gut was evident and appeared to coincide with bacterial transition into coccoid forms. At this time point, a significant reduction in the overall density of *B*. *bacilliformis* per fly was observed, concurrent with erythrocyte digestion by both *L*. *verrucarum* and *L*. *longipalpis*. Although colonization of both sand fly species exhibited reduction in bacterial density at the 72-h time point, *B*. *bacilliformis* appeared at relatively high densities in locations beyond the abdominal midgut in *L*. *verrucarum* such as the thoracic midgut and ileum (Fig 4A–4D).

In general, this was not the case for *L*. *longipalpis* at 72 h post-blood meal (Fig 4E–4H). Although bacteria were occasionally observed beyond the AM, densities were relatively lower and the decreased size suggested the bacteria may not have been viable. Thus, at 72 h post-blood meal, a difference was observed between the competent and non-competent vectors of *B*. *bacilliformis*

**Fig 5:**
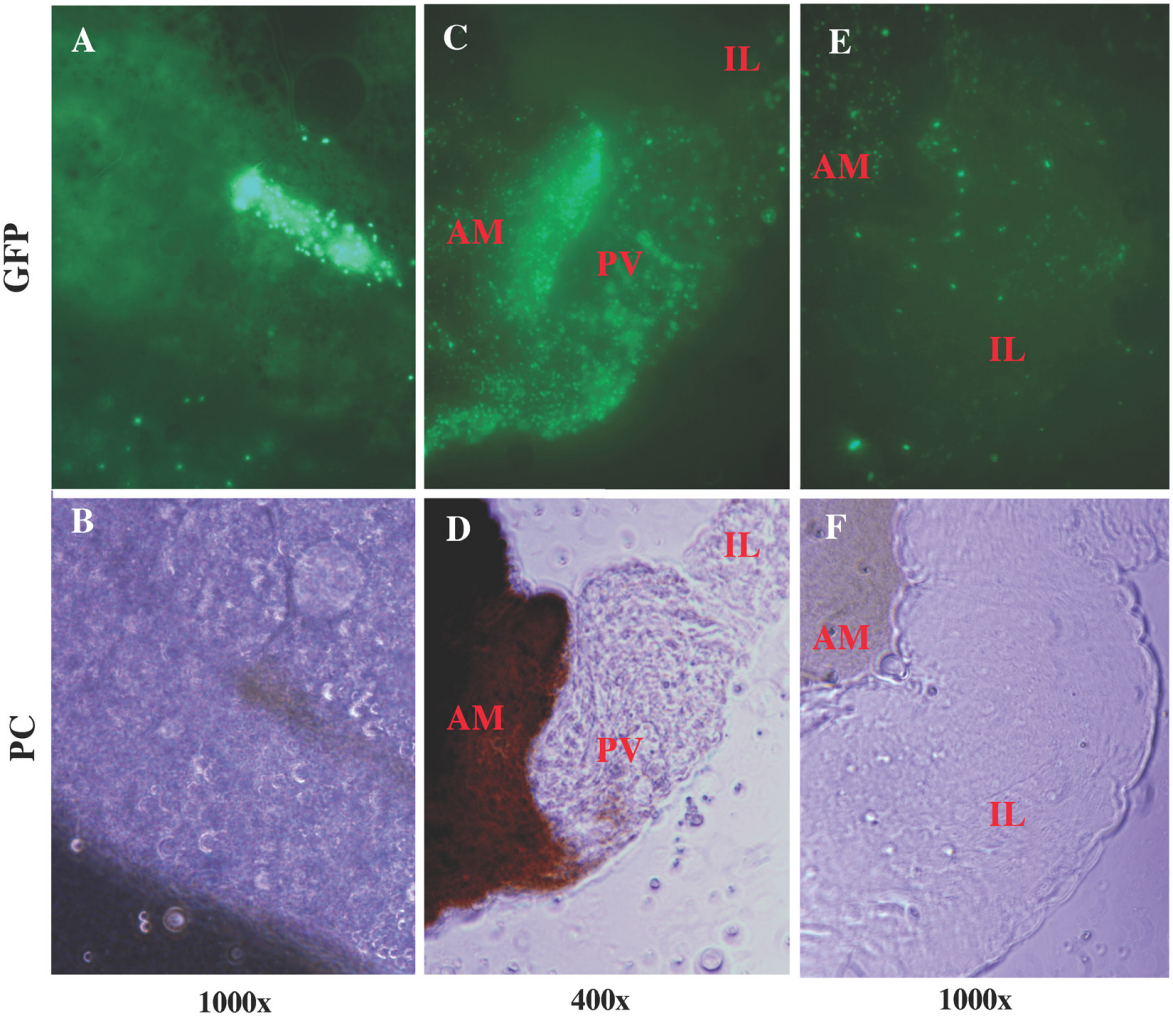
*L*. *verrucarum* at 5d post-blood meal. After 5 d *B*. *bacilliformis* colonized the competent sand fly digestive tract. As observed at 72 h following the blood meal, bacteria were found in the abdominal midgut **(A-B)** as well as the ileum (**C-F**). Images were acquired with fluorescence (GFP) or phase contrast (PC) microscopy and the magnification used is indicated under each image pair.

**Fig 6:**
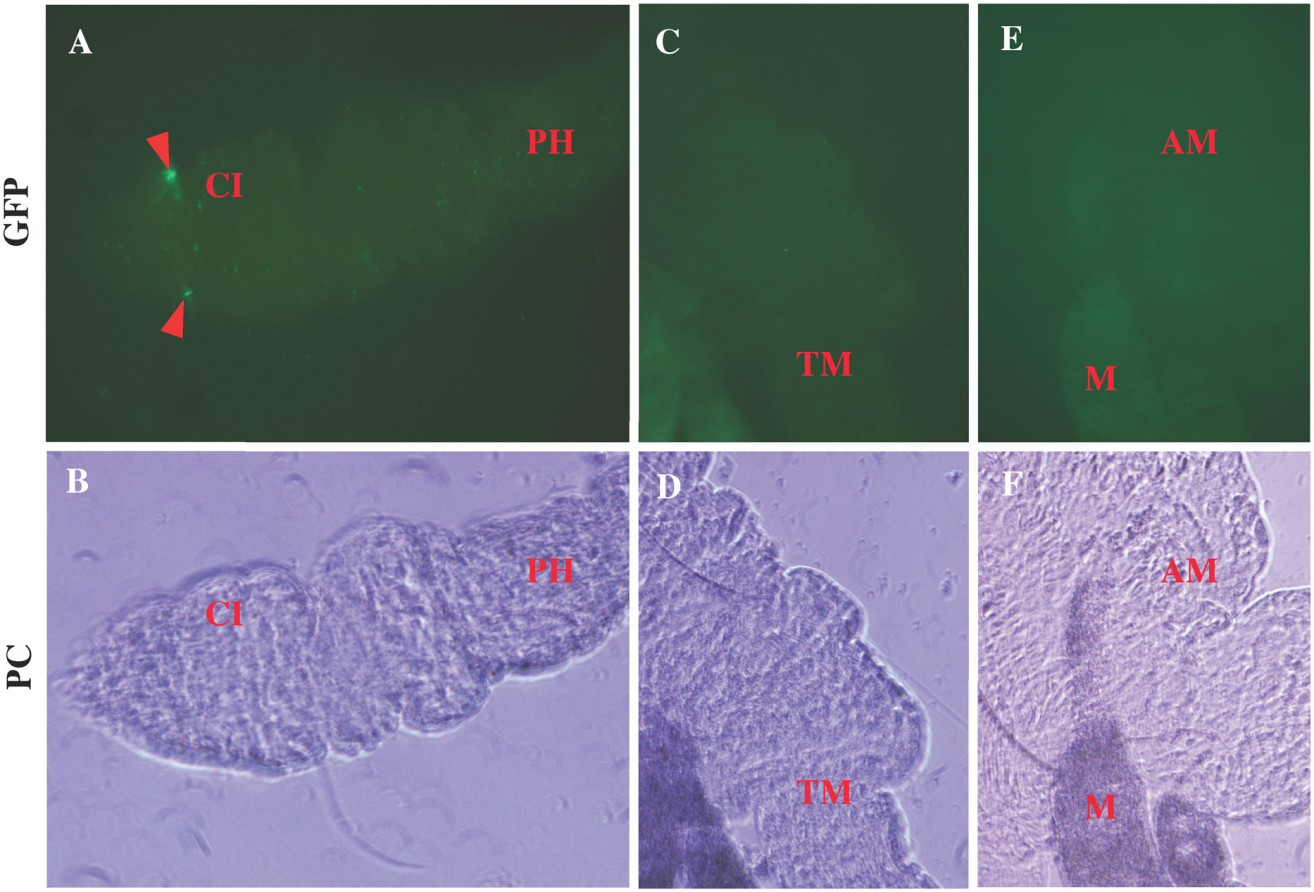
*L*. *longipalpis* at 7d post-blood meal. A small number of bacteria were observed (arrowheads) in the cibarium (CI), which is immediately connected to the proboscis and pharynx (PH) **(A-B)**. Observation of *B*. *bacilliformis* in *L*. *longipalpis* tissues other than the abdominal midgut was rare, and samples of the non-competent vector continued to demonstrate digestion/disappearance of bacteria over time **(C-F)**. Images acquired with fluorescence (GFP) or phase contrast (PC) microscopy at 400x magnification.

At 5 d post-blood meal, the *L*. *verrucarum* digestive tract continued to be colonized by *B*. *bacilliformis* beyond the boundary of the AM (Fig 5). Colonization of *L*. *longipalpis* examined at this time point exhibited a significant decrease in density, where minute GFP signals, and questionably viable bacteria, were observed. One example of a questionable signal, however, was observed in the cibarium of *L*. *longipalpis* at 7 d post-blood meal (Fig 6). Thus, by 7d post-blood meal, *B*. *bacilliformis* was apparently cleared from *L*. *longipalpis*, whereas infection of *L*. *verrucarum* was quite apparent.

**Fig 7:**
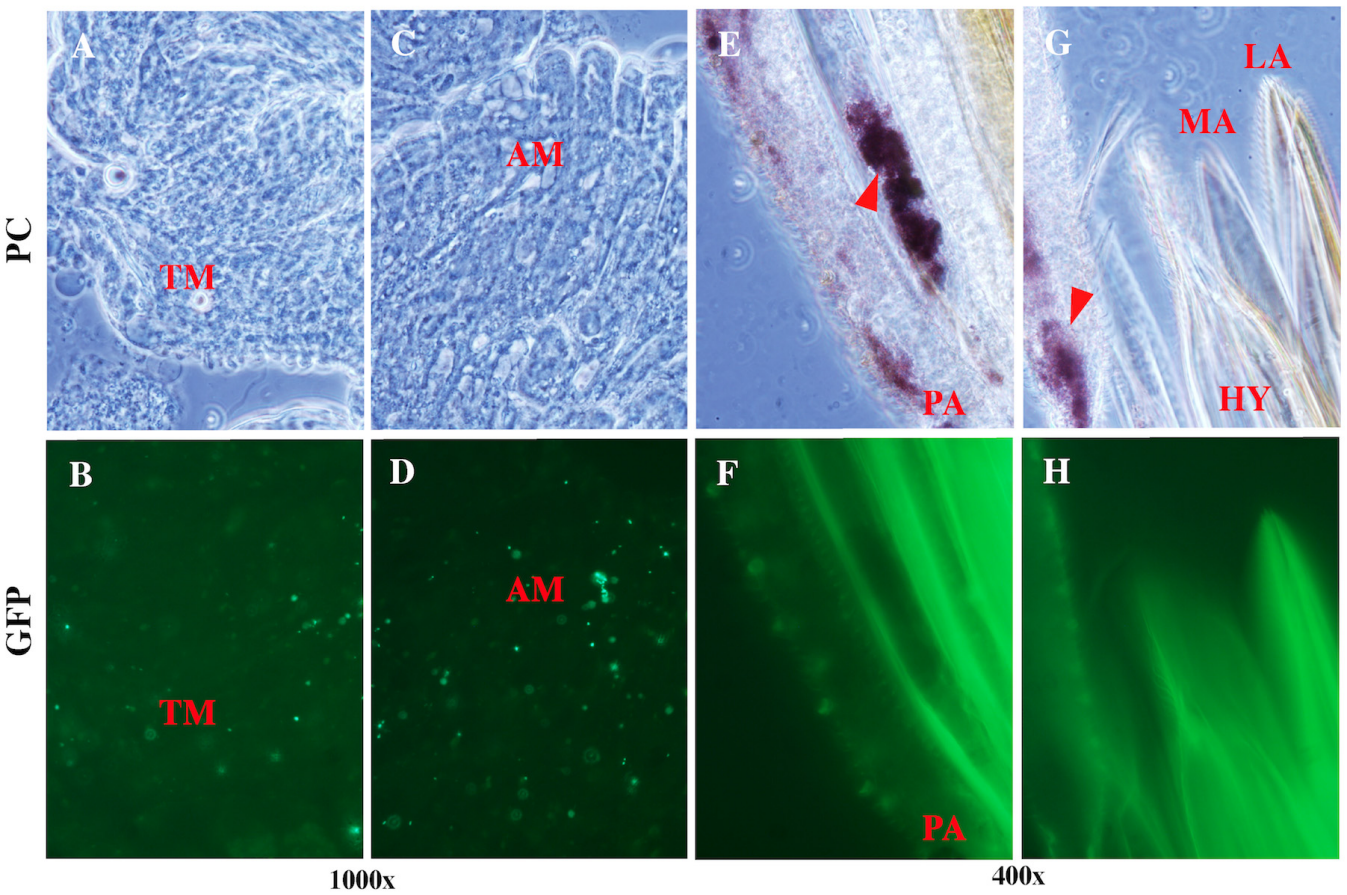
*L*. *verrucarum* at 14d post-blood meal. Two weeks after competent sand flies imbibed infected blood, *B*. *bacilliformis* colonization persisted, but was limited to the digestive tract **(A-D)**. We were unable to observe GFP^+^ bacteria on mouthparts **(E-H)** but relatively large brown-colored coccoid forms were present (arrowhead). Perhaps this is what Hertig referred to as organism *x-prob* (*see* S1 Fig).

At 14 d post-blood meal, three *L*. *verrucarum* were analyzed and one of the flies had an obvious infection of the anterior and thoracic midgut (TM; Fig 7A–7D). Fly 2 had one GFP^+^ signal in its ilieum, and the third fly had no signal whatsoever.

Overall, microscopic analyses comparing competent and non-competent sand fly vectors suggest that *L*. *longipalpis* eliminates *B*. *bacilliformis*, whereas in *L*. *verrucarum*, bacteria survive blood meal digestion, migrate beyond the AM, and colonize the sand fly’s entire digestive tract. We were unable to observe *B*. *bacillliformis* outside the digestive tract lumen. We were also unable to visualize GFP-expressing bacteria in the feces or diuretic fluid of *L*. *longipalpis*. Unfortunately, we were unable to corroborate the feces and diuretic fluid data with *L*. *verrucarum* due to limited availability of this sand fly species.

### Viability of *B*. *bacilliformis* in *L*. *longipalpis*

**Fig 8:**
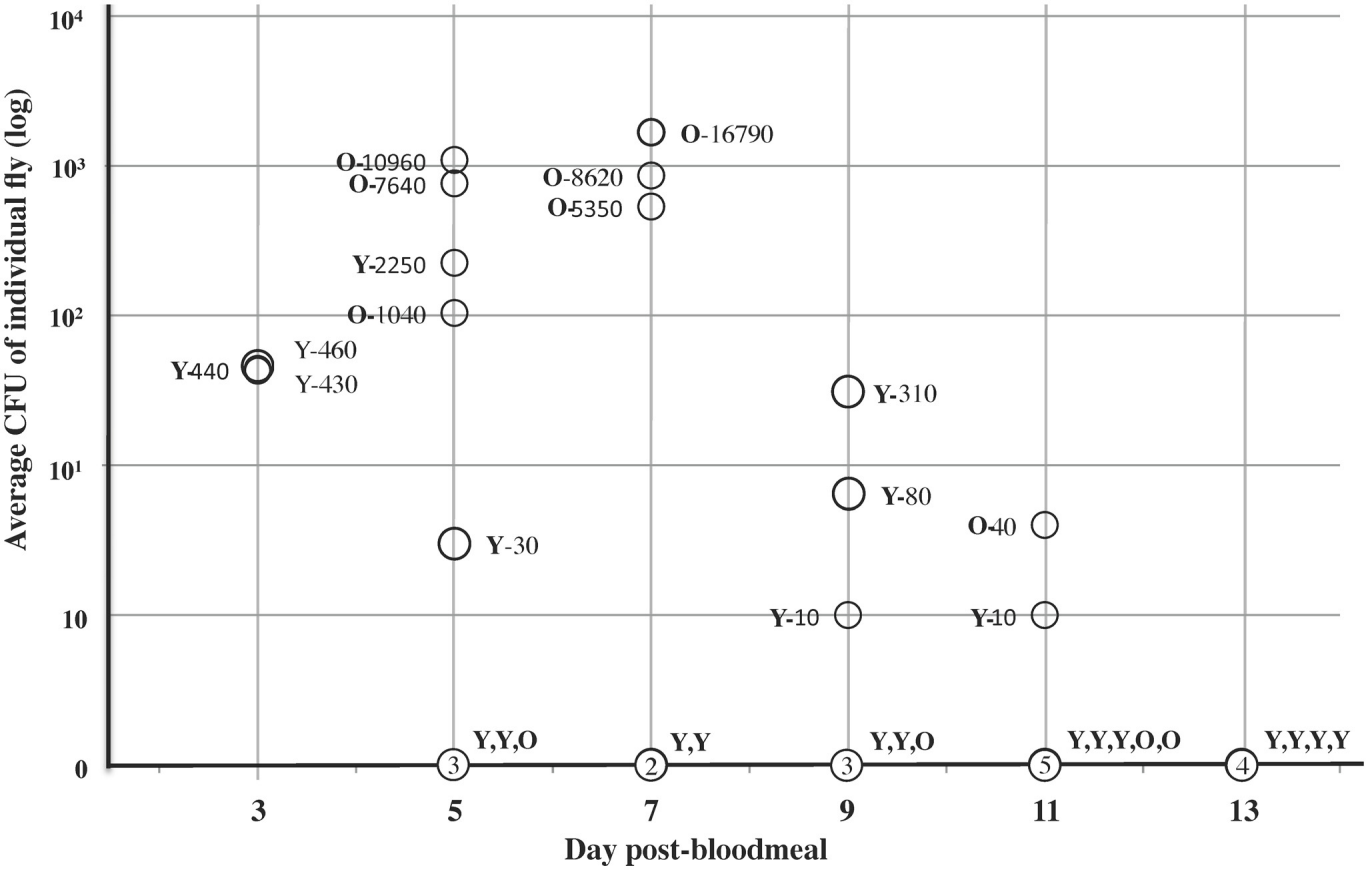
Viability of *B*. *bacilliformis* in *L*. *longipalpis* adults over time. Four groups of 200 sand flies were offered *B*. *bacilliformis-*infected blood meals (6x10^6^ bacteria/ml) and average colony forming units (CFUs) were determined over a 13-d time course and are plotted in log scale. Average numbers of bacteria per individual fly are provided, along with the age group where young and old are indicated (Y, O). Flies that have apparently cleared the infection are also shown, with the number of flies circled and age group indicated at each time point.

In this experiment, we analyzed the viability of *B*. *bacilliformis* in both young (2–10 d) and old (11–25 d) sand flies and were able to make several observations (Fig 8). First, *B*. *bacilliformis* remained viable in the non-competent vector for up to 11d post-infection. Second, older flies appeared to maintain a higher density of bacteria over time compared to younger flies. Third, by 13 d post-infection, viable *B*. *bacilliformis* were no longer found in *L*. *longipalpis*.

### Mechanical Transmission of *B*. *bacilliformis* by *L*. *longipalpis*

We were curious whether *L*. *longipalpis* could transmit viable *B*. *bacilliformis* from an artificially-infected blood meal offered through a chick-skin membrane on a glass feeder to an uninfected blood meal offered through a chick-skin membrane on an identical feeder simply by means of contaminated mouthparts. However, groups of 50 and 150 sand flies were unable to transmit bacteria from the infected blood meal to the uninfected blood meal, suggesting *L*. *longipalpis* was unable to transmit viable *B*. *bacilliformis* by mechanical means.

Hertig examined wild-caught *L*. *verrucarum* by light microscopy, and cultured *B*. *bacilliformis* from proboscis homogenates [18]. In addition, he frequently observed ‘massive infections of the sand fly proboscis with unidentified microorganisms’, and termed these *‘x-prob’*. He was unable to culture *‘x-prob’*. In our study, we frequently observed something similar to ‘*x-prob’* on the mouthparts of laboratory-reared *L*. *verrucarum* as well as *L*. *longipalpis* (S1 Fig). However, we were unable to observe GFP signal on mouth parts of infected flies at any point over the 14-d time course. This indicates that ‘*x-prob’* is distinct from *B*. *bacilliformis*. Thus, we were curious if these unknown bodies contained DNA, and were actually microorganisms. We collected 10–12 d-old *L*. *longipalpis* that had not been exposed to *B*. *bacilliformis* and attempted to stain putative *‘x-prob’* with common nucleic acid stains (e.g., ethidium bromide, SYTO9 and propidium iodide) without success (S1D and S1E Fig). Given the lack of DNA and striking resemblance between these structures and eye pigmentation granules, we hypothesize that they are not bacteria but rather pigment granules from omatidia in the compound eyes.

## Discussion

This study represents the first microscopic characterization of *B*. *bacilliformis* colonizing the midgut of *Lutzomyia* sand flies since 1942. We used fluorescence microscopy to chronicle colonization of *L*. *verrucarum* and *L*. *longipalpis* by using a low-passage GFP-expressing *B*. *bacilliformis* isolate over a 14-d time course. Our results suggest that *B*. *bacilliformis* infects both species of sand fly, and colonization is limited to the lumen of the digestive tract. As infection progresses, bacteria appear to change morphology from a highly motile bacillus into a relatively smaller, non-motile coccoid form. A similar phenomenon was previously described for *B*. *bacilliformis* cultured in vitro; wherein the bacterium changed from a motile bacillary form into a non-motile coccoid form upon entering stationary phase [19].

When examined microscopically, differences in the course of colonization between sand fly species were indistinguishable up to 72h following an infected blood meal. At this time point, *B*. *bacilliformis* appeared to transform into small coccoid forms, that by 7d post-blood meal were nearly absent from *L*. *longipalpis*. However, at 72h post-blood meal, *B*. *bacilliformis* appeared at relatively high density in regions of the gastrointestinal track beyond the abdominal midgut and persisted for >14d as small coccoid forms. Although bacteria exhibited a significant degree of motility at 24h post-infection, by 48 h-post infection *B*. *bacilliformis* appeared to bind to the peritrophic membrane and were immobile in both fly species. Although a small number of bacteria were observed outside the PM, it is unclear whether this occurred prior to or after formation of the PM.

As mentioned, Carrión’s disease is not a zoonosis, but rather an anthroponosis, as a non-human animal reservoir of *B*. *bacilliformis* has never been identified. Transovarial transmission is a phenomenon in which a pathogen is maintained in nature by arthropods, where eggs are infected and subsequently maintained in the vector until the adult stage, when transmission to human occurs. Transovarial transmission of Rio Grande Virus [20] is known to occur in *Lutzomyia* and we were curious if this could occur with *B*. *bacilliformis*. We therefore examined eggs (n >100) of both species inside the fly and following oviposition, but were unable to observe GFP^+^ bacteria in the eggs of either fly species. Furthermore, eggs collected from infected *L*. *longipalpis*, were cultured on HIB-B+Kan plates but viable *Bartonella* CFUs were never isolated. These results suggest that maintenance of *B*. *bacilliformis* in nature is not facilitated by transovarial transmission.

At dusk, humans enter dwellings to sleep and sand flies become active and seek blood. Humans generally feel pain associated with the ‘bite’ of a sand fly (which mechanically is a pierce and cut rather than a ‘bite’) and attempt to slap the affected area. This reaction either results in the fly’s escape (to seek more blood) or its demise at or near the bite region on the surface of skin. A phenomenon referred to as ‘mechanical transmission’ or early phase transmission (EPT) [21] is one in which arthropod mouthparts are initially contaminated, by briefly obtaining small amounts of blood from an infected human, and then finishing its meal on another human. Our interrupted blood meal EPT experiments with *B*. *bacilliformis* by relatively large populations of *L*. *longipalpis* suggest that mechanical transmission by this species does not occur. Whether EPT occurs during a bite with contaminated *L*. *verrucarum* is unknown but would likely prove interesting.

Noguchi examined a wide variety of arthropods from the verruga zone (e.g., ticks, mites, midges, lice, fleas, bedbugs, buffalo gnats, mosquitoes, horse flies, sheep ticks, and three species of sand fly) that were collected without the use of chemicals and shipped to Rockefeller Institute [11]. Each arthropod was subsequently crushed in saline and injected into rhesus monkeys. The only samples that generated a *B*. *bacilliformis* bacteremia were of *L*. *noguchii* and *L*. *verrucarum*, while samples of *L*. *peruensis* or of any of the other arthropods generated no infection. Battistini collected live infected sand flies from the verruga zone that were released into a screened cage containing rhesus monkeys and *B*. *bacilliformis* was subsequently isolated from the blood of these animals [22]. This was apparently the first demonstration of *B*. *bacilliformis* transmission by sand flies. It is unclear whether transmission occurred from ‘biting’ or some other means.

It is well accepted that *Bartonella quintana* transmission to humans is primarily a result of the infected feces of *Pediculus humanus* (human body lice) being scratched into a cutaneous bite. The resulting infection causes trench fever [23]. In 1937, Hertig described an experiment wherein wild *L*. *verrucarum* were fed on patients that were bacteremic and 75% of 90 flies contained *B*. *bacilliformis* in their guts when later examined [24]. Interestingly, fecal matter obtained from these infected sand flies contained a large number of *B*. *bacilliformis*, whereas diuretic fluid did not. Unfortunately, we were unable to analyze feces or diuretic fluid of *L*. *verrucarum* to any great extent due to limited numbers of this sand fly species. However, samples of *L*. *longipalpis* feces and diuretic fluid examined were devoid of any visible GFP signal or viable bacteria.

Finally, Hertig attached small cages containing infected sand flies to Rhesus monkeys and, although transmission to animals occurred, he mentioned that it may have been by some other means than direct inoculation via mouthparts [18]. We have demonstrated that large groups of *L*. *longipalpis* do not appear to mechanically transfer viable bacteria as a ‘flying needle’, but because *B*. *bacilliformis* remains viable for up to seven days, this species may be capable of transmission by other means.

In conclusion, our results characterize colonization of the sand fly midgut by *B*. *bacilliformis* as well as demonstrate a distinct pattern regarding colonization of competent and non-competent arthropod species. We have shown *L*. *longipalpis* to be a user-friendly, live-vector/host model system, which we currently use to further characterize site-specific mutants of *B*. *bacilliformis* in *Lutzomyia*. Identification of factors used by pathogens to colonize and persist in arthropod vectors may lead to the development of transmission-blocking vaccines [25] [26] for prevention of this severely neglected tropical disease.

## Supporting Information

S1 Video *L*. *verrucarum* abdominal midgut at 24h post-blood meal. Bacteria exhibited a significant degree of motility. Colonization of *L*. *verrucarum* at this time point was strictly limited to the abdominal midgut, encased in a peritrophic membrane along with the blood meal. Video acquired at 1000x.
(MOV)

S2 Video *L*. *longipalpis* abdominal midgut at 24h Post-Blood meal. Bacteria exhibited a significant degree of motility. As observed *in L*. *verrucarum*, colonization of *L*. *longipalpis* after 24h was limited to the abdominal midgut. Video acquired at 1000x.
(MOV)

S3 Video *L*. *verrucarum* abdominal midgut at 48h post-blood meal. *B*. *bacilliformis* morphology was smaller coccoid form and appeared more abundant than at 24h time point. Motility was significantly decreased. Bacteria appeared to adhere to the peritrophic matrix of both species of sand fly. Colonization of *L*. *longipalpis* by *B*. *bacilliformis* at this time point was indistinguishable from *L*. *verrucarum*.
(MOV)

S1 Fig *L*. *verrucarum* at 14d post-blood meal. Two weeks after competent sand flies imbibed an infected blood meal, *B*. *bacilliformis* colonization persisted, but was limited to the digestive tract. **(A)** In 1942 Hertig described massive infections of the proboscis of wild-caught sand flies with unidentified microorganisms he distinguished from *B*. *bacilliformis* and termed *x-prob*. He was unable to culture *x-prob* and injection of organism in animals yielded no infection. Here, we viewed what is likely *x-prob* **(B)** by phase contrast and **(C)** flourescence microscopy and verified that no GFP signal is was associated with this organism. Last, we used a number of nucleic acid stains to label DNA of the suspected microorganism, without success. Phase contrast **(D)** and flourescence microscopy **(E)** indicated no signal when stained for 15 min with ethidium bromide. Collectively, these data suggest *x-prob* was not a microorganism, but rather eye pigmentation granules. Panel A (Panel 9 from [18]) reprinted by permission of American Journal Tropical Medicine and Hygiene.
(TIF)

S1 Data Materials and methods for mass rearing sand fly colonies. Materials and methods regarding mass rearing of sand flies is not novel. However, we provided details used in this study for ease of replication or follow-up work including specifics on housing, feeding and animal use.
(PDF)
