# Supplementary figures and images for: Colonization of Lutzomyia verrucarum and Lutzomyia longipalpis Sand Flies (Diptera: Psychodidae) by Bartonella bacilliformis, the Etiologic Agent of Carrión’s Disease
Source: PLoS Negl Trop Dis. 2015 Oct 5;9(10):e0004128. doi: 10.1371/journal.pntd.0004128 (PMC4593541; doi:10.1371/journal.pntd.0004128)

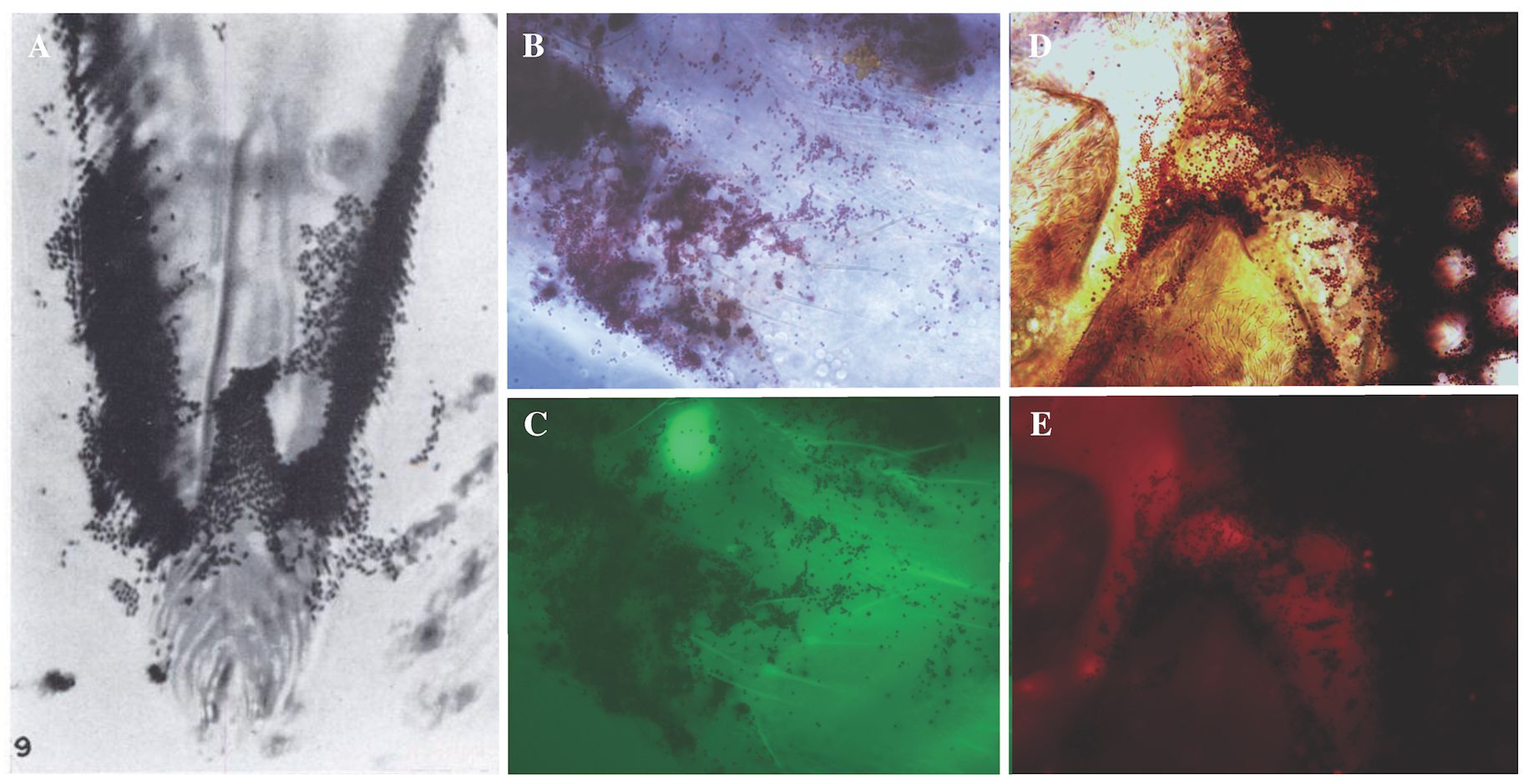

Supplement: S1 Fig — Two weeks after competent sand flies imbibed an infected blood meal, B. bacilliformis colonization persisted, but was limited to the digestive tract. (A) In 1942 Hertig described massive infections of the proboscis of wild-caught sand flies with unidentified microorganisms he distinguished from B. bacilliformis and termed x-prob. He was unable to culture x-prob and injection of organism in animals yielded no infection. Here, we viewed what is likely x-prob (B) by phase contrast and (C) flourescence microscopy and verified that no GFP signal is was associated with this organism. Last, we used a number of nucleic acid stains to label DNA of the suspected microorganism, without success. Phase contrast (D) and flourescence microscopy (E) indicated no signal when stained for 15 min with ethidium bromide. Collectively, these data suggest x-prob was not a microorganism, but rather eye pigmentation granules. Panel A (Panel 9 from [18]) reprinted by permission of American Journal Tropical Medicine and Hygiene. (TIF) [file pntd.0004128.s004.tif]
