# Supplementary material for: Colonization of Lutzomyia verrucarum and Lutzomyia longipalpis Sand Flies (Diptera: Psychodidae) by Bartonella bacilliformis, the Etiologic Agent of Carrión’s Disease
Source: PLoS Negl Trop Dis. 2015 Oct 5;9(10):e0004128. doi: 10.1371/journal.pntd.0004128 (PMC4593541; doi:10.1371/journal.pntd.0004128)
Supplement: S1 Data — Materials and methods regarding mass rearing of sand flies is not novel. However, we provided details used in this study for ease of replication or follow-up work including specifics on housing, feeding and animal use. (PDF) [file pntd.0004128.s005.pdf]

Supporting Data S1

Sand Fly Mass Rearing

Immature stages were reared in custom made 'larval pots' consisting of modified 500-ml polycarbonate jars (Nalgene) with 6-8 holes (1.6 cm dia.) drilled in the bottom and overlaid with 2.54 cm of Plaster of Paris (POP). Lids of the jars were modified by drilling a 2.54 cm hole in the center and covering it with cloth mesh (CM; ~52 squares/2.54 cm) to allow for gas exchange. Prior to use, vented lids and jars were cleaned with sterile water, the POP was hydrated by soaking overnight in sterile water, and wiped dry with autoclaved paper towels. Larval pots were stored in autoclaved polypropylene boxes containing water-soaked sponges at 25˚C (80-90% RH) in a climate-controlled incubator (Percival Scientific; Perry, IA) within the insectary.

Typically, ~150 blood-fed females were transferred to larval pots at 24h following a blood meal on anesthetized mice (below). Each fly subsequently layed 30-35 eggs on the POP surface that hatched within 10 d. Larval food consisted of a composted 1:1 mixture of rabbit feces and rabbit chow (LabDiet 5325; Purina, Wilkes-Barre, PA). Small amounts of food were gently sprinkled on the POP surface for the 40 to 60-d time period (depending on species) for development through the adult stage.

Anesthetized Mice Provide Blood Meals for Generation of Eggs

Rearing a sand fly colony for experimental use requires continuous propagation wherein one third of the adult population is used for research and the remaining two thirds is used to perpetuate the colony. In this study, *L. longipalpis* was reared by providing female sand flies with anesthetized mice (Laboratory Animal Resources, University of Montana) as a source of blood. Two to four mice were anesthetized with 0.1 ml /10 g body weight of anesthetic (0.3 ml of 100 mg/ml ketamine; 0.2 ml 20 mg/ml xylazine and 2 ml sterile PBS [pH 7.4]), eyes protected with salve and placed in adult mating/holding cages. An aspirator was used to exhale CO_2 into the chamber to stimulate feeding and flight. Adult flies and anesthetized mice were placed in the refrigerated incubator (25˚C; 80-90% RH) for 30-45 min. In general, 3-4 four mice fed approximately 1000 flies. All experiments described were performed following approval by the Institutional Animal Care and Use Committee (University of Montana).

Blood-fed flies were held in the cage overnight (25˚C; 80-90% RH) to allow for diuresis and hardening of the peritrophic membrane and then were transferred to an ‘ova pot’ to lay eggs.

Emergent adults were released into custom-made 30.5 cm^3 holding/mating cages (21st Century Plastics, Missoula, MT). Cages were cleaned with 70% ethanol prior to each use.
